# Cold atmospheric plasma increases IBRV titer in MDBK cells by orchestrating the host cell network

**DOI:** 10.1080/21505594.2021.1883933

**Published:** 2021-02-08

**Authors:** Yujie Miao, Peiyu Han, Dong Hua, Renwu Zhou, Zhengbing Guan, Qing Lv, Xiaofeng Dai

**Affiliations:** a Wuxi School of Medicine, Jiangnan University, Wuxi, China; b The Key Laboratory of Industrial Biotechnology, Ministry of Education, School of Biotechnology, Jiangnan University, Wuxi, China; c Affiliated Hospital of Jiangnan University, Wuxi China; d School of Chemical and Biomolecular Engineering, University of Sydney, Sydney, Australia

**Keywords:** Cold atmospheric plasma, IBRV, MDBK, host-virus interaction

## Abstract

Enhancing virus multiplication could assist in the rapid production of vaccines against viral diseases. Cold atmospheric plasma (CAP), a physical approach relying on reactive oxygen species to achieve the desirable cellular outcome, was shown to be effective in enhancing virus propagation, where bovine rhinotrachieitis virus and Madin-Darby Bovine Kidney cells were used as the modeling virus and cell line, respectively. CAP was shown to create synergies with virus infection in arresting host cells at the G2/M stage, decreasing cell membrane potential, increasing intracellular calcium level, and inducing selective autophagy. In addition, CAP was demonstrated to suppress virus-triggered immunogenic signaling as evaluated by IRF7 expression. We presented evidences on CAP-triggered maximization of host resources toward virus multiplication that is advantageous for viral vaccine production, and opened a novel regime for applying CAP in the sector of medical care and health.

## Introduction

Vaccine has long been considered as an effective approach for viral disease prevention and control, especially during an epidemic outbreak such as Coronavirus Disease 2019 (COVID-19) that is caused by Syndrome Coronavirus 2 (SARS-CoV-2) and currently threatening global health [1]. Increasing virus titer is an important strategy to meet the high demand for vaccine production that utilizes cells for virus manufacturing.

Cold atmospheric plasma (CAP), the fourth state of matter composed of reactive oxygen and nitrogen species (RONS) such as hydroxyl radical (OH**·**), hydrogen peroxide (H_2_O_2_), ozone (O_3_), superoxide anion (O^2·-^), nitric oxide (NO·), and electron (e^−^) [2], is a dose-dependent physical approach capable of achieving diverse cell responses [3,4]. Low to medium doses of CAP could subject cells to programmed events such as cell cycle arrest, senescence, autophagy, apoptosis, and immunogenic cell death [3]. Of particular interest here is the possible trigger of cell cycle arrest by CAP that has been reported almost 10 years ago [5], as virus infection could alter cell cycle toward arrest at G_2_/M, G_1_/S and G_0_/G_1_ phases to favor virus multiplication [6]. This makes CAP a possible tool to boost viral particle yield by setting cells at the basal metabolic level that maximizes cell resources for virus production.

Motivated as such, we explored the efficacy of CAP in boosting virus titration and synergies between virus infection and CAP in hijacking host cells toward virus multiplication. Using infectious bovine rhinotrachieitis virus (IBRV) and Madin-Darby Bovine Kidney (MDBK) cells as the models of virus and host cell, respectively, we investigated how CAP modulates the host cell machinery toward orchestrated networking favorable for virus production. Following modulated events on viral infection as previously identified [6], we examined how CAP modulated calcium (Ca^2+^) influx, autophagy, and immune response in addition to cell cycle to gain a full picture of CAP-triggered cell network alteration*. Our study makes CAP a potential tool for rapid viral vaccine production and opens a novel research field for physical plasma application in medicine.

## Methods

### CAP ejection source and preparation

The CAP ejection device as well as the operation procedure were shown in supplementary Figure 1a. The experimental setup for CAP generation is comprised of a power controller, helium (He) gas cylinder, rotor flow meter, and plasma jet. The peak-to-peak voltage applied to the electrode was set in the range of 0.96–1.24 kV, the sinusoidal wave frequency was set to 10 kHz, the flow rate of He gas was set to 1 L/min, and the distance between the plasma source and the medium surface was fixed to 13 mm. The CAP-activated medium was generated by exposing 1 mL medium to CAP treatment for 4 min for each well in 24-well plates.

**Figure 1.**
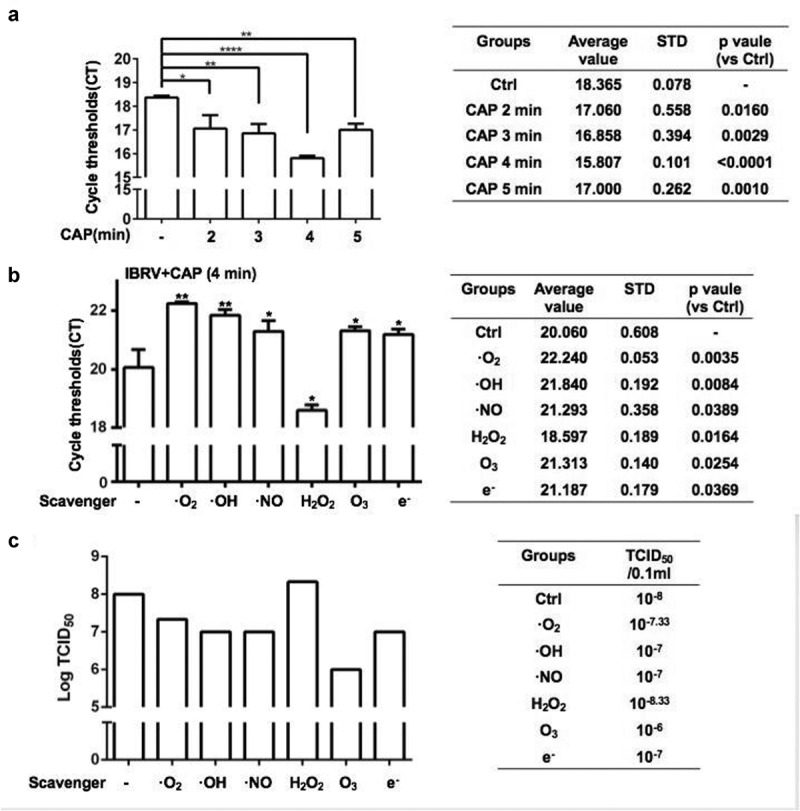
**IBRV titer in response to CAP exposure** (a) **at different doses, and** (b, c) **under different ROS quenchers as measured from** (a, b) **q-PCR and** (c) **TCID50**. Hydroxyl radical (OH**·**), hydrogen peroxide (H_2_O_2_), ozone (O_3_), superoxide anion (O^2·-^), nitric oxide (NO·), and electron (e^−^) are quenched by mannitol, uric acid, tiron, hemoglobin, and monopotassium phosphate, respectively

### Optical emission spectroscopy (OES) of CAP

Reactive oxygen and nitrogen species in the gas phase CAP were detected using a spectrometer (Andor Shamrock SR-500i-A-R, England). The optical probe was placed at a distance of approximately 10 mm from the center of the CAP ejection device.

### Cells and viruses

The MDBK cell line was purchased from the Cell Bank of the Chinese Academy of Sciences Cell Bank and cultured in RPMI medium containing 10% fetal bovine serum (FBS) and 1% penicillin-streptomycin. IBRV (NCBI TaxId: 79,889) was purchased from the Type Culture Collection of the Chinese Academy of Sciences, Shanghai, China and propagated using MDBK cells.

### MDBK cell infection

MDBK cells were grown to 80%-90% confluence followed by IBRV inoculation. Cells were harvested 48 h after IBRV infection.

### TCID50 assay

Isolated IBRV venom was subsequently diluted to 10-folds (10^−1^ to 10^−10^) and inoculated to MDBK cells that had grown to 80%–90% confluence in 96-well microculture plates, with 8 wells for each dilution fold and 100 μL for each well. Cells without virus inoculation were set as the control. Cells were incubated at 37°C in 5% CO_2_ for 1 h followed by cultivation using low serum DMEM medium (2.5% serum) at 37°C in a 5% CO_2_ incubator for CPE observation. CPE was observed under a microscope for 5 consecutive days, and TCID50 was calculated by Reed–Muench method.

## RT-PCR

Supernatants (samples) were collected following the manufacture’s protocol of UltraSYBR MixTure Kit (CW0957M, Cwbio Co. Ltd.). 4 μL samples, 10 μL 2×UltraSYBR MixTure, 1 μL forward and 1 μL backward primers, 4 μL ddH_2_O were mixed and centrifuged before running the RT-PCR program (pre-denature at 95°C for 10 min; 95°C for 10 sec, 60°C for 1 min, 72°C for 20 sec, for 40 cycles) in Roche LightCycler 480 RT-PCR. Each sample had three replicates. Primers used in this study are listed in Table 1.

**Table 1.** Primers for IBRV, *IRF7*, and *IRF3.*

Groups	Forward Primer	Reverse Primer
IBRV	CGTGGTGGTGCCAGTTAG	TCATCGTCGCTGTCGTCAT
*IRF3* (NM_001029845.3)	ACCCAACACTTAGGCCAGAC	TGGGCTCAAGTCCATGTCAC
*IRF7* (NM_001105040.1)	CAGTGACCACGGAGGGC	GCATCTTCTAGGGCCTCGTC

### Western blot

MDBK cells were grown in six-well plates, washed twice with PBS, lysed on ice for 20 min using RIPA lysis buffer containing a protease inhibitor, and centrifuged at 12,000 g for 20 min before collecting the supernatants. Protein concentration was estimated using a BCA Proteib analysis kit (Beyotime). The protein per lane (50 μg) was resolved by SDS-PAGE and transferred to a PVDF membrane. After blocking with 5% skim milk powder in TBS and Tween-20 buffer, the membrane was incubated with a suitable primary antibody at 4°C overnight, and then incubated with the secondary antibody for 1 h at room temperature. By developing the blot using an enhanced chemiluminescent reagent, antibody binding was observed. Bands were visualized using OmegaLumG (UVP) and analyzed using Image J software. The total protein of infected MDBK cells was extracted, separated using an 8% SDS-PAGE gel at 110 V for 80 min, and transferred from the SDS-PAGE gel to a PVDF membrane using a membrane transfer device. After being incubated at 4°C for 8–12 h and washed with TBST, a primary antibody was added. The secondary antibody was replenished and incubated with cells at room temperature for 1–2 h after TBST cleaning and gel imager detection.

### Immunofluorescence imaging

Cells were plated in a 96-well plate filled with complete DMEM containing 1% antibiotics and 10% (v/v) FBS, and cultured at 37°C, 5% CO_2_ and saturated humidity until cells reached 80–90% confluence. The culture medium was discarded and cells were washed three times with PBS. The remaining liquid was aspirated and discarded, and cells were fixed at the room temperature for 15 min. The fixative was discarded, and cells were washed three times with PBS. 1 mL of PBS+0.5% TritonX-100 was added to cells, and the mixture was set on ice for 5 min. The permeabilization solution was discarded, and cells were washed three times with PBSB (PBS+5% BSA). 1 mL PBSB was added and cells were blocked with slow shaking at room temperature for 30 min. The blocking solution was discarded and cells were washed three times with PBST (PBS+0.5% Tween-20 buffer). The primary antibody was diluted with PBSB, and 300 μL diluted primary antibody was added to cover the slide followed by slow shaking overnight at 4°C. The primary antibody was recovered and cells were washed three times with PBST. The fluorescent secondary antibody was diluted with PBSB, 300 μL of which was supplemented to the well to cover the glass slide followed by incubation at room temperature for 1 h in the darkness. The secondary antibody was recovered and cells were washed 3 times with PBST in the darkness. DAPI was diluted with PBSB (1:200), 300 μL of which was added to wells to stain the nucleus slowly for 1 min in the darkness. The nucleus staining solution was exhausted and cells were washed three times with PBST in the darkness. Tweezers were used to gently remove the glass slide, and the remaining liquid was drained using the filter paper. About 5 μL of 50% glycerin was added, and samples were stored in the darkness. Photoes were taken using Zessi 780 laser confocal microscope.

### Membrane potential

Cells were plated in a 96-well plate filled with complete DMEM containing 1% antibiotics and 10% (v/v) FBS, and cultured at 37°C, 5% CO_2_, and saturated humidity until cells reached 80–90% confluence. The DiBAC4(3) (Bis-(1,3-Dibutylbarbituric Acid)Trimethine Oxonol) mother solution was diluted at a ratio of 1:1000, and added to MDBK cells grown in a 96-well microculture plate at a volume of 50 μL/well followed by incubation at 37°C, 5% CO_2_, and saturated humidity for 30 min. A full-wavelength microplate reader was used to measure the signals with the excitation wavelength set to 388 nm and the emission wavelength set to 418 nm.

### Ca^2+^ level examination

Cells were plated in a 96-well plate filled with complete DMEM containing 1% antibiotics and 10% (v/v) FBS, and cultured at 37°C, 5% CO_2_, and saturated humidity until cells reached 80–90% confluence. The FURA-2-AM CELL PERMEANT reagent was diluted at a ratio of 1:1000, and added to MDBK cells grown in a 96-well microculture plate at a volume of 50 μL/well followed by incubation at 37°C, 5% CO_2_, and saturated humidity for 30 min. A full-wavelength microplate reader was used for signal measurement where the excitation wavelength was set to 380 nm and the emission wavelength was set to 510 nm.

### Flow cytometry

MDBK cells were grown in 6-well plates, washed with PBS, and digested with EDTA-free trypsin. After removing the supernatants, cells were centrifuged at 1000 rpm/min for 5 min to retain the pellets. Cell pellets were washed with 500 μL of PBS, centrifuged at 1000 rpm/min for 5 min, and the supernatant was removed to retain the pellet. Cell pellets were resuspended in 70% ethanol and placed in a refrigerator at 4°C overnight for fixation. Fixed cells were centrifuged at 1000 rpm/min for 5 min to remove the supernatant, and cell pellets were suspended in 500 μL of PBS. 5 μL of propidium iodide (purchased from Beyotime) stain was added and mixed with cells on ice for 30 min. Cell cycle detection was performed using a BD Accuri C6 flow cytometer, and data analysis was performed using Flowjo software.

### ROS scavenger assay

Cells were plated in a 96-well plate filled with complete DMEM containing 1% antibiotics and 10% (v/v) FBS, and cultured at 37°C, 5% CO_2_, and saturated humidity until cells reached 80–90% confluence. About 10 μL of sodium pyruvate (100 mM), uric acid (1 mM), mannitol (2 M), Tiron (200 mM), hemoglobin (200 μM), monopotassium phosphate (10 mM) was added to 1 ml of CAP-treated medium, each at one time, before inoculating cells with the virus and performing RT-PCR.

## Results

### CAP increases IBRV titer through ROS-induced G2/M cell-cycle arrest

**Figure 2.**
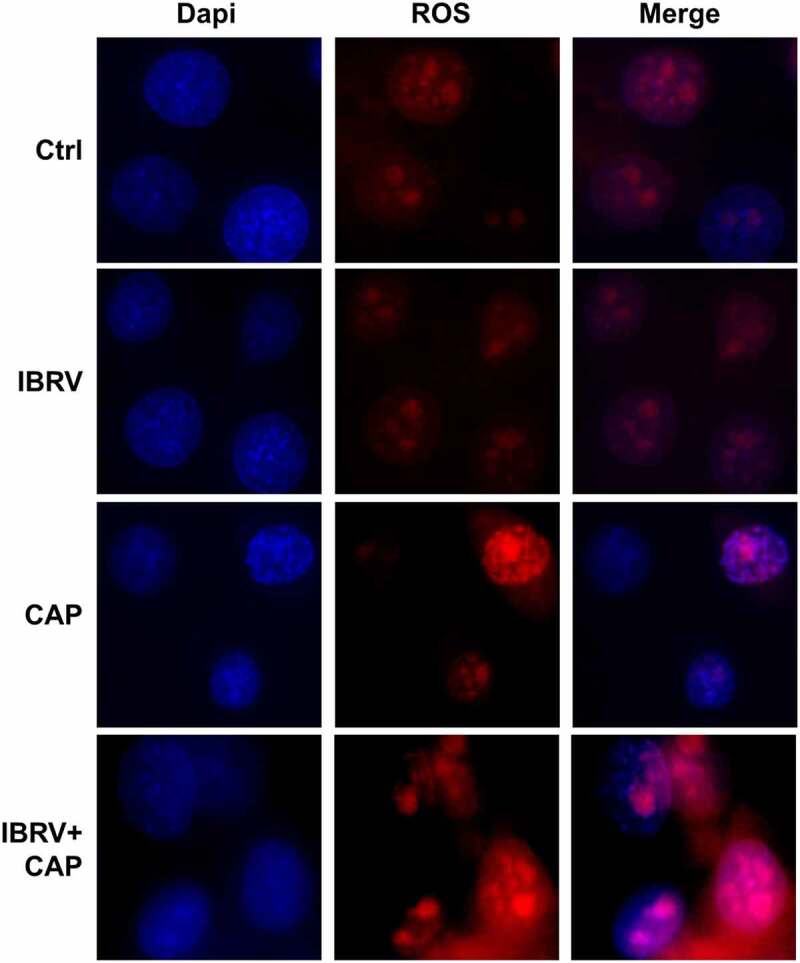
Comparisons on cell redox level in cells on IBRV infection and/or CAP exposure

**Figure 3.**
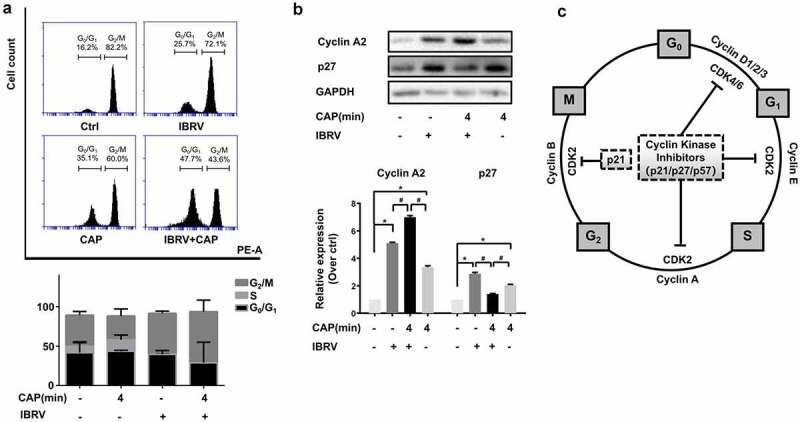
**Cell cycle arrest of IBRV-infected cells on IBRV infection and/or CAP exposure**. (a) Cell cycle profiles from cell cytometry and their quantifications. (b) Western blot on key cell cycle molecules, and (c) their roles in cell cycle progression

When being exposed to CAP, IBRV titration significantly increased with the optimal yield being achieved at the 4-min dose (CAP exposure for 4 min; p < E-4 for 4-min dose, Figure 1a). The highest yield was 10E-5.5 TCID50/0.1 mL, over 10 folds that of the control (supplementary Figure 2a). The chemical spectrum of CAP in the gas phase was composed primarily of OH that may form H_2_O_2_ in the liquid phase (supplementary Figure 1b). By quenching the activity of each typical ROS component, we found that the virus yield could be further enhanced if H_2_O_2_ was removed and short-lived species such as O^2-^, OH, .NO, e^−^, and the long-lived species O_3_ all played important roles in mediating the observed effect on increasing virus titer (Figure 1b, Figure 1c). Removing reactive species from CAP, each at one time, decreased virus titer except for H_2_O_2_ that significantly (p = 0.0164) decreased the qPCR cycle threshold (Figure 1b) and enhanced virus titer from 10E-8 to 10E-8.33 TCID50/0.1 mL (Figure 1c, supplementary Figure 2b). Enhanced reactive oxygen species (ROS) were detected in IBRV-infected cells on CAP exposure (Figure 2). ROS overproduction is known to induce G2/M cell-cycle arrest [7,8]. In consistent with this, we found that the cell cycle was tiltered toward G2/M enrichment on synergistic exposure to CAP and IBRV (Figure 3a). Accordingly, Cyclin A2 expression was elevated and its inhibitor p27 was lowered (Figure 3b), which together promoted cells to pass through the S phase toward G2/M cell cycle arrest (Figure 3c).

### CAP creates synergies with IBRV infection through triggering Ca^2+^ influx

**Figure 4.**
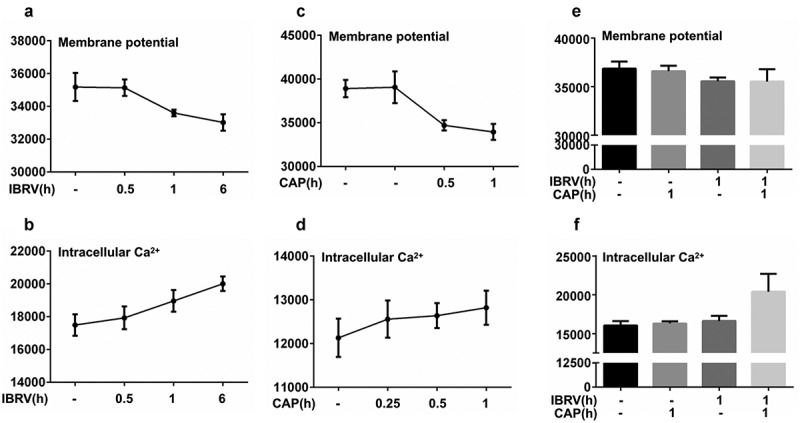
**Cell membrane potential and intracellular Ca^2+^ level on IBRV infection and/or CAP exposure**. (a) Cell membrane potential and (b) intracellular Ca^2+^ level under different IBRV incubation durations. (c) Cell membrane potential and (d) intracellular Ca^2+^ level under different CAP exposure durations and follow-up cultivation procedures. Synergies between CAP exposure and IBRV infection on (e) cell membrane potential and (f) intracellular Ca^2+^ level. (g) Immunofluorescence imaging showing Ca^2+^ influx on IBRV infection and/or CAP exposure

Cell membrane potential decreased and intracellular Ca^2+^ level increased with the duration that cells were incubated with IBRV (Figure 4a, Figure 4b). The profiles of cell membrane potential and intracellular Ca^2+^ level after IBRV infection were consistent with those of cells exposed to CAP treatment for different durations, i.e. cell membrane potential decreased and intracellular Ca^2+^ level increased with CAP exposure durations (Figure 4c, Figure 4d). Joint exposure of cells to CAP and IBRV decreased cell membrane potential (Figure 4e) and increased intracellular Ca^2+^ level with statistical significance as compared with administrating CAP or IBRV alone (p = 5.5E-3, figure 4f). Immunofluorescence imaging showed cellular accumulation of Ca^2+^ on joint CAP and IBRV exposure, suggesting a significant Ca^2+^ influx (Figure 4g). These results were consistent with our understandings of the intrinsic connections between Ca^2+^ influx and cell cycle, i.e. increased Ca^2+^ influx is associated with altered cell shape and promotes cell cycle toward mitogenesis [9–12].

### CAP creates synergies with IBRV infection through inducing selective autophagy

**Figure 5.**
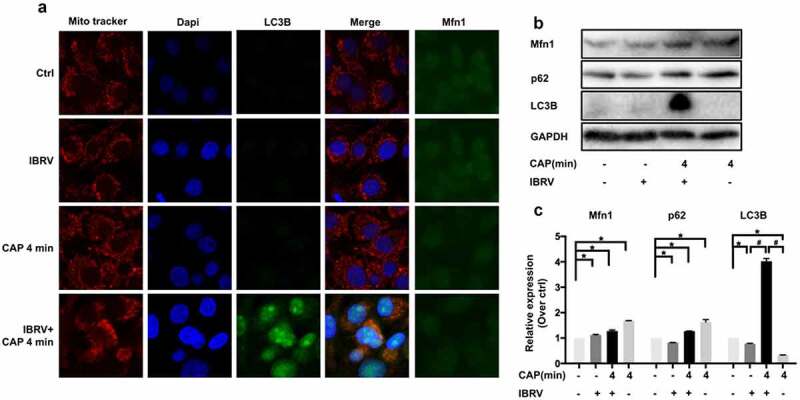
**Comparisons on selective autophagy in cells on IBRV infection and/or CAP exposure**. (a) Immunofluorescence imaging. (b) Western blot on key proteins in selective autophagy and (c) their quantifications

Selective autophagy is featured by the binding of LC3/GABARAP interacting region (LIR) containing proteins such as SQSTM1/p62, NBR1, NDP52, and BNIP3 to a specific autophagy substrate and tethering the substrate to the site of autophagosomal engulfment through interactions between LIR containing proteins and autophagosome-specific proteins such as LC3 family members [13–15]. LC3B was overexpressed and colocalized with mitochondria on CAP exposure in IBRV-infected cells (Figure 5a), suggesting the existence of selective autophagy.

Mitochondrial turnover depends on autophagy, where p62 binds voltage-dependent anion-selective channel protein (VDAC) and MFN1/2 (mitochondrial outer membrane GTPases mediating mitochondrial clustering and fusion) to the mitochondria toward ubiquitination and mitophagic degradation [16]. MFN1 was overrepresented on joint CAP and IBRV exposure (Figure 5b, Figure 5c), suggesting an economic use of mitochondria and the existence of mitochondrial recycling to sustain virus multiplication.

### CAP creates synergies with IBRV infection in suppressing immunogenic signaling

**Figure 6.**
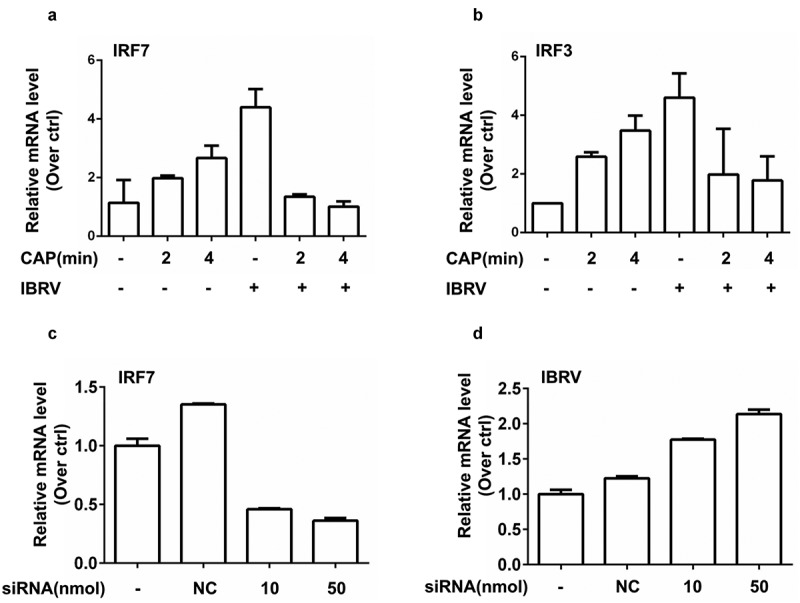
**Expression of key genes in immunogenic signaling and their effects on IBRV multiplication**. (a) IRF7 and (b) IRF3 gene expression on IBRV infection and/or CAP exposure. (c) Knocking down efficiency of IRF7, and (d) its effect on IBRV multiplication

IFN regulatory factor 3 (IRF3) and IRF7 are two primary transcription factors inducing type I IFNs (IFNα, IFNβ) production that could prime host cells for T helper 1 adaptive immune response against virus invasion [17–21]. Either CAP exposure or IBRV infection triggered IRF3 and IRF7 overexpression; yet CAP significantly reduced the increased level of IRF7 on IBRV infection back to normal (p = 0.021 for IRF7 at 2-min CAP, p = 0.018 for IRF7 at 4-min CAP, Figure 6a), and suppressed IRF3 expression with marginal significance (p = 0.171 for IRF3 at 2-min CAP, p = 0.077 for IRF3 at 4-min CAP, Figure 6b). Different from IRF3 that is constitutively expressed, IRF7 expression entirely depends on type I IFN signaling and is affected by viral infection [18]. Knocking down *IRF7* (p < E-4 for 10 nm siRNA, p = 3E-4 for 50 nm siRNA, Figure 6c, Table 2) significantly increased viral DNA production (p = 1.4E-3 for 10 nm siRNA, p = 0.029 for 50 nm siRNA, Figure 6d, Table 2), suggesting that suppressed immunogenic signaling as represented by IRF7 was favorable for virus multiplication.

**Table 2.** SiRNA sequences for knocking down *IRF7.*

	SiRNA primer sequences targeting IRF7
Forward	GCUCUUCGGAGACUGGCUUTT
Reverse	AAGCCAGUCUCCGAAGAGCTT

## Discussion

**Figure 7.**
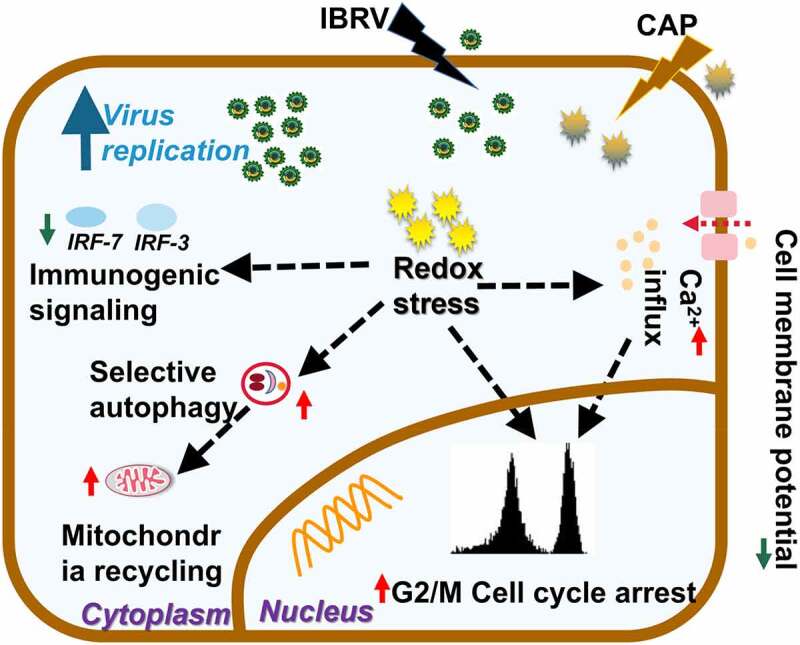
**Conceptual illustration on cell orchestrated processes favoring virus multiplication on joint IBRV infection and CAP exposure**. By imposing cellular redox stress, CAP triggers DNA damage signaling that leads to G2/M cell cycle arrest and enhanced selective autophagy including mitophagy. CAP could also induce lipid peroxidation that is accompanied with Ca^2+^ influx and decreased cell membrane potential. While these alterations prepared favorable intracellular environment for virus multiplication, CAP suppresses immunogenic signaling to protect host cells from immune recognition that establishes a homeostatic and friendly extracellular environment for virus to propagate

CAP was found to assist IBRV in creating a favorable environment for virus multiplication via promoting cell cycle arrest at the G2/M stage, triggering Ca^2+^ influx, inducing selective autophagy, and suppressing immunogenic signal generation as a result of cellular redox modulation (Figure 7). By imposing cellular redox stress, CAP triggers DNA damage signaling that leads to G2/M cell cycle arrest and enhanced selective autophagy including mitophagy. CAP could also induce lipid peroxidation that is accompanied by Ca^2+^ influx and decreased cell membrane potential. While these alterations prepared a favorable intracellular environment for virus multiplication, CAP suppresses immunogenic signaling to protect host cells from immune recognition that establishes a homeostatic and friendly extracellular environment for viruses to propagate.

G2/M cell cycle arrest is the most relevant biological process to cell proliferation [22]. It is a typical cell behavior in response to DNA damage signaling and subjected to checkpoint regulation where, in this context, elevated cellular ROS as triggered by CAP imposed the cellular stress. Cells prepare lots of DNA and proteins required for mitogenesis when passing through the S phase and arriving at the G2/M stage, and halted mitogenesis makes it possible for viruses to hijack these cell resources for virus multiplication.

Ca^2+^ influx is essential for G1 progression [9–11], promoting the synthesis of RNA and ribosomes at the G1 stage that constitutes the halt of cells at the G2/M stage. It was reported that lipid peroxidation increased Ca^2+^ permeability of hepatocyte plasma membrane [23]. Thus, the observed cell cycle arrest might be triggered by lipid oxidative stress in response to ROS-mediated lipid peroxidation.

Oxidative stress typically results in mitochondrial malfunction, and selective autophagy removes dysfunctional organelles including mitochondria that drives mitochondrial turnover and is indispensable for malfunctional organelle recycling [24]. Elevated selective autophagy and shrinked use of mitochondria for cell growth on CAP exposure in IBRV-infected cells (Figure 5) implicated the maximization of cell resources for virus multiplication at the expense of minimizing resources for cell survival.

Compromised immunogenic signaling protects virus-infected cells against immune cell recognition and thus is favorable for virus propagation. We found that CAP reduced the enhanced level of IRF3/7 on IBRV infection while increasing IRF3/7 in normal cells (Figure 6a, Figure 6b). On the other hand, ROS has emerged as important players in regulating innate immunity, and H_2_O_2_ was shown capable of creating synergies with herpes simplex virus 2 (HSV-2) in elevating the expression of type I IFNs [25]. Interestingly, by quenching H_2_O_2_ the efficacy of CAP in boosting virus titer was further enhanced (Figure 1b). Thus, though H_2_O_2_ is an important long-lived component of CAP, it could not represent the full profile of CAP that produces both long- and short-lived reactive species such as singlet oxygen [26]. In addition, the efficacy and outcome of CAP is mediated by interactions between CAP components and cell surface characteristics such as catalysis and SOD expression that are essential for H_2_O_2_ cell entry [27]. Thus, it was possible that IBRV entry modified cell surface and thus reduced H_2_O_2_ entry, and the other components of CAP-triggered cellular signaling that led to reduced IRF3/7 expression. Though enhanced CAP efficacy as a result of quenching H_2_O_2_ provides certain experimental evidence, this hypothesis is, however, subjected to rigorous experimental tests before being considered true.

Though these events are all associated with the oxidative nature of CAP, no CAP component could replace CAP in boosting virus titer, as CAP components may interact with each other and with cell surface to initiate an avalanche of cell signaling that could not be simplified as just oxidation.

### Conclusion

We demonstrated in this paper that CAP could halt the cell cycle at the G2/M stage and trigger selective autophagy to maximize cell resources for virus multiplication, and suppress immunogenic signaling as represented by IRF3/7 expression to maintain an environment favorable for host survival. Thus, CAP represents a potential tool for enhancing vaccine production against viral diseases that deserves intensive efforts to explore mechanisms driving its synergies with virus infection.

## Supplementary Material

Supplemental Material
