# Supplementary material for: Cold atmospheric plasma increases IBRV titer in MDBK cells by orchestrating the host cell network
Source: Virulence. 2021 Feb 8;12(1):679–89. doi: 10.1080/21505594.2021.1883933 (PMC7889027; doi:10.1080/21505594.2021.1883933)
Supplement: Supplemental Material [file KVIR_A_1883933_SM8951.docx]

**Supplementary figure legends**

**
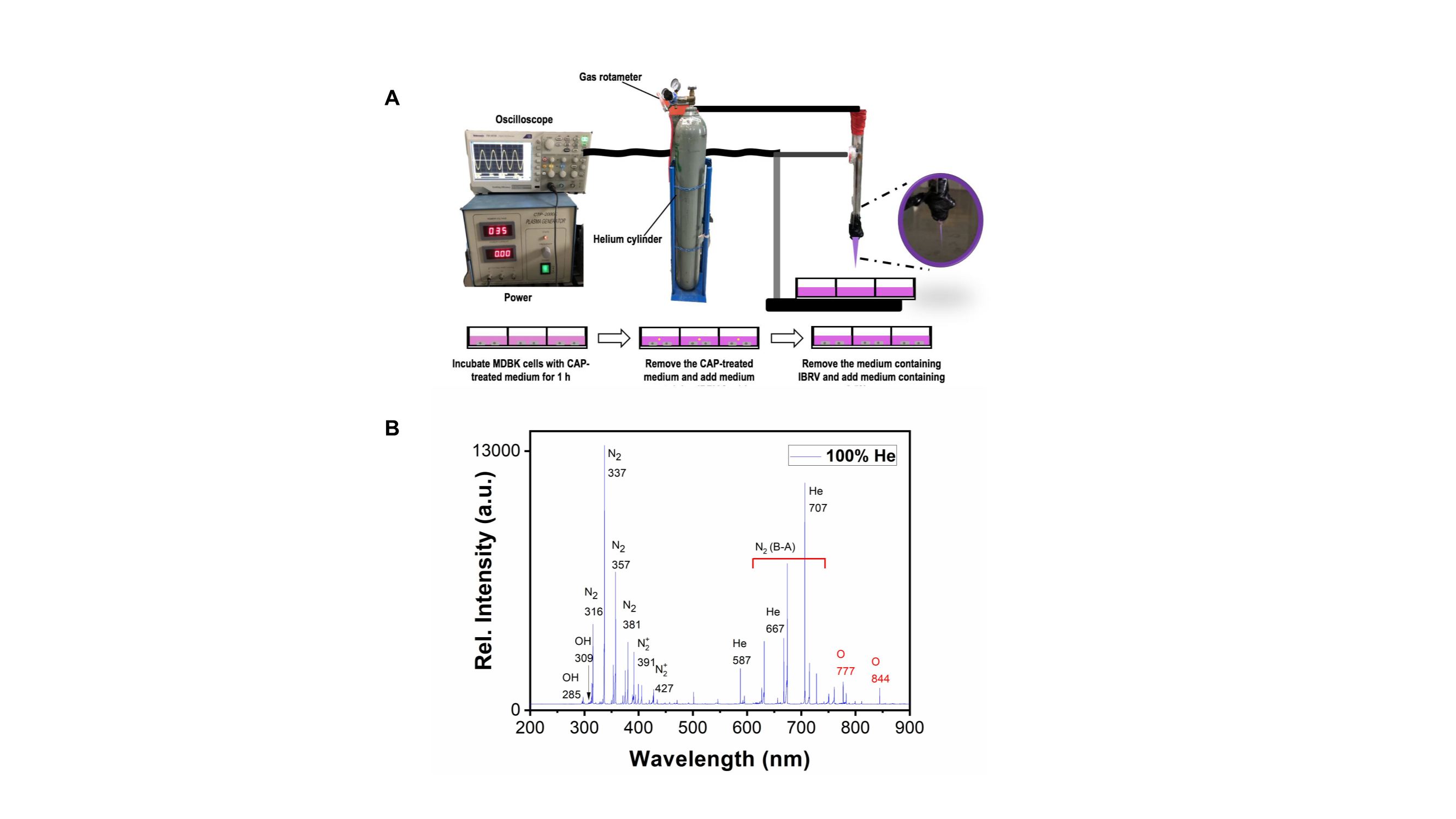
**

**Supplementary Figure 1. Cold atmospheric plasma ejection device configuration, experimental design and cold atmospheric plasma component analysis. (A)** Cold atmospheric plasma ejection device configuration and experimental design. **(B)** Optical emission spectroscopy of CAP ejected from the presented device.

**
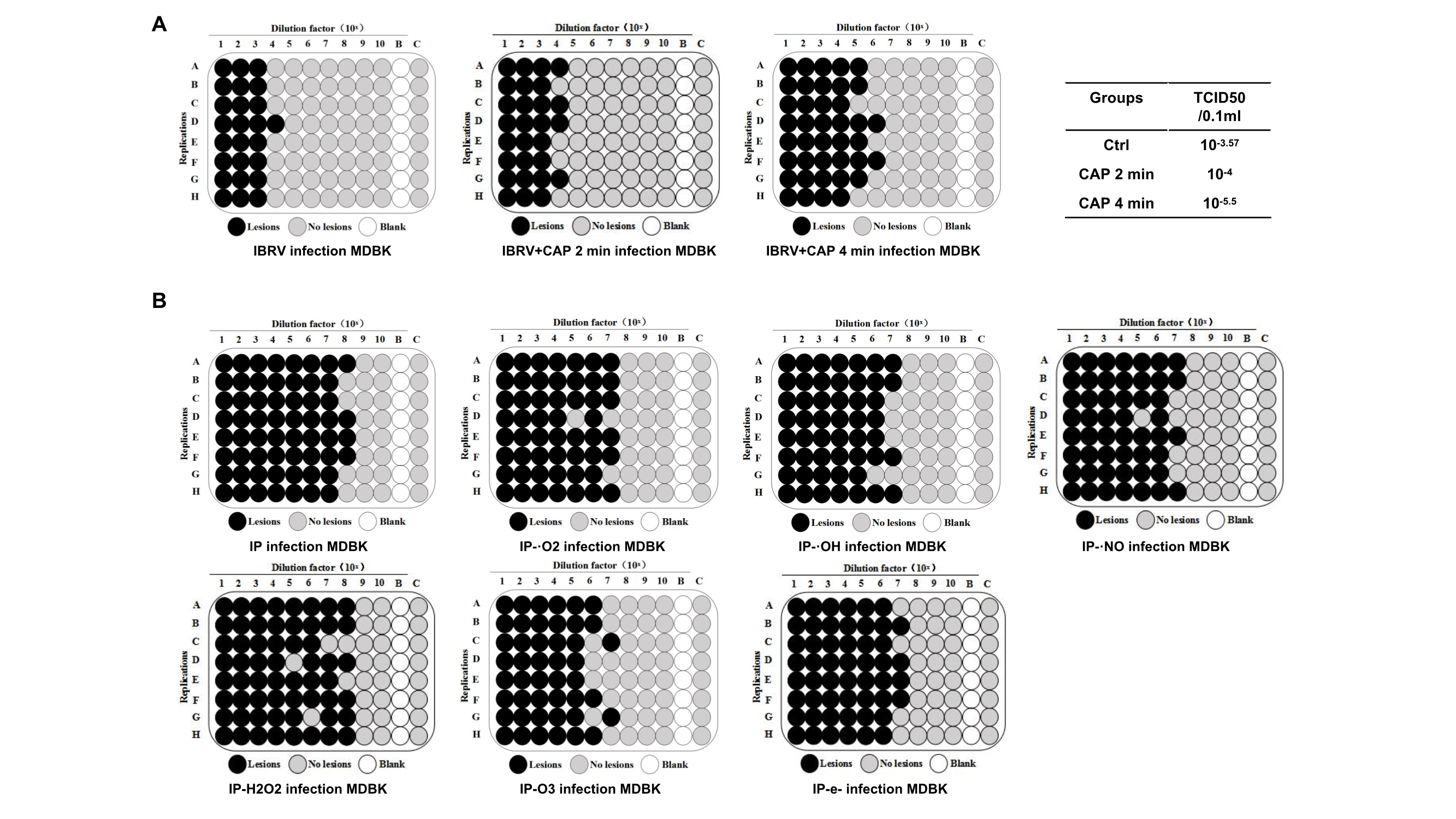
**

**Supplementary Figure 2. Raw TCID50 experimental results on IBRV titer in response to CAP exposure (A) at different doses, and (B) under different ROS quenchers.** Hydroxyl radical (OH**·**), hydrogen peroxide (H_2_O_2_), ozone (O_3_), superoxide anion (O^2·-^), nitric oxide (NO·), and electron (e^-^) are quenched by mannitrol, uric acid, tiron, hemoglobin and monopotassium phosphate, respectively.
